# Low-Temperature PLD-Growth of Ultrathin ZnO Nanowires by Using Zn_*x*_Al_1−*x*_O and Zn_*x*_Ga_1−*x*_O Seed Layers

**DOI:** 10.1186/s11671-017-1906-2

**Published:** 2017-02-20

**Authors:** Alexander Shkurmanov, Chris Sturm, Helena Franke, Jörg Lenzner, Marius Grundmann

**Affiliations:** Felix Bloch Institute for Solid State Physics, Universität Leipzig, Linnéstr. 5, Leipzig, 04103 Germany

**Keywords:** ZnO nanowires, Ultrathin nanowires, High-pressure PLD

## Abstract

ZnO nanowires (NWs) are used as building blocks for a wide range of different devices, e.g. light emitters, resonators, and sensors. Integration of the NWs into such structures requires a high level of NWs’ diameter control. Here, we present that the doping concentration of Zn_*x*_Al_1−*x*_O and Zn_*x*_Ga_1−*x*_O seed layers has a strong impact on the NW growth and allows to tune the diameter of the NWs by two orders of magnitude down to less than 7 nm. These ultrathin NWs exhibit a well-oriented vertical growth and thus are promising for the investigation of quantum effects. The doping of the ZnO seed layers has also an impact on the deposition temperature which can be reduced down to *T*≈400^∘^C. This temperature is much smaller than those typically used for the fabrication of NWs by pulsed laser deposition. A comparison of the NWs indicates a stronger impact of the Ga doping on the NW growth than for the Al doping which we attribute to an impact of the size of the dopants. The optical properties of the NWs were investigated by cathodoluminescence spectroscopy which revealed a high crystalline quality. For the thin nanowires, the emission characteristic is mainly determined by the properties of the surface near region.

**Electronic supplementary material:**

The online version of this article (doi:10.1186/s11671-017-1906-2) contains supplementary material, which is available to authorized users.

## Background

The fabrication of nanostructures such as nanowires (NWs) attracted a lot of interest in the last years, since they allow to reduce the size of devices, e.g. light emitters [1], electromechanical resonators [2], and the realization of devices with high spatial resolution, e.g. pressure and 3D imaging sensors [3, 4]. The desired geometry of the NWs is determined by the applications. For example, thick NWs with a thickness of several hundreds of nanometer up to few micrometer are preferred for applications which based on the compression of the NWs, e.g. caused by an externally applied pressure, whereas thin NWs (few nanometer thick) are interesting for surface and bending sensitive applications [5]. These wires are interesting also for gas sensors due to their large surface-to-volume ratio [6]. For example, an enhanced sensitivity of thin NWs to oxygen was reported by Fan and Lu [7]. Of special interest are ultrathin NWs with a diameter comparable to the Bohr radius. For such thin ZnO NWs, a dipole moment of about 10 times larger than that in bulk crystal was predicted by Dag et al. [8]. Furthermore, the presence of quantum effects in these wires is expected, which would opens new fields of applications, e.g. formation of topological qubits [9, 10].

However, there are only a limited number of reports which present the growth of ultrathin NW, e.g. by using electro-chemical deposition [11], metalorganic vapor phase epitaxy deposition [12, 13], carbothermal growth [14], and hydrothermal growth method [15]. The drawback of these methods is that they require catalysts which can be partly incorporated into the NWs and might have an influence on their optical and electrical properties. Moreover, NWs grown by these techniques are typically not well-oriented. These drawbacks can limit the usability of the NWs and the perfomance of devices. A promising method to overcome the mentioned drawbacks, i.e. the growth of well-oriented verticaly aligned NWs without using a catalyst, is pulsed laser deposition (PLD). The growth of NWs by PLD was introduced about one decade ago [16] and is now a well-established technique [17]. This growth represents a trade-off between simplicity and controlling of the NWs’ properties [16–18]. However, the obtained NWs have typically a diameter between 60 and 600 nm and thus are much thicker than the Bohr radius of the exciton. Here, we show that the composition of the underlying seed layer has a significant impact on the diameter of the NWs. By using Al- and Ga-doped ZnO seed layers, we were able to achieve well-oriented NWs with diameters less than 7 nm. Furthermore, we show that the choice of the seed layer has also a strong impact on the growth temperature which can be strongly reduced from *T*≈950^∘^C, typically used during the PLD process, down to *T*≈400^∘^C. This relatively low process temperature can preserve CMOS structure [19] and thus high-crystalline ZnO can be integrated in these structures by using this growth process.

## Methods

In order to investigate the NW growth as a function of the Ga and Al concentration of the seed layer, we deposited Zn_**x**_Al_1−**x**_O and Zn_**x**_Ga_1−**x**_O thin films with 0≤*x*≤7 at.% on *a*-plane sapphire substrate by using a conventional low-pressure PLD process. Thereby, we use an oxygen partial pressure of about *p*=0.01 mbar and a growth temperature of about 720^∘^C. The sintered ceramic targets used for the growth of the seed layers were produced by a mixture of ZnO and Al_**2**_O_**3**_ or rather Ga_**2**_O_**3**_ powders. The concentration of the dopants in the thin films was measured by energy-dispersive X-ray spectroscopy (EDX) analysis and the concentration was determined to be 0≤*x*≤7 at.%. Note that in the case of the Zn_**x**_Al_**1−x**_O films, the measurements were performed on reference films grown on Si (100) substrates deposited under the same conditions as the seed layers for the NW growth in order to avoid an incorrect determination of the Al concentration by the sapphire substrate. All seed layers have a thickness of about 200−250 nm which was determined by spectroscopic ellipsometry.

**Fig. 1:**
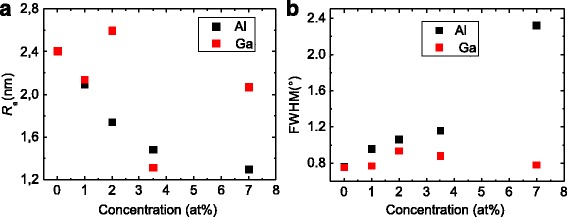
Surface roughness (**a**) and the FWHM of rocking curves (**b**) of the seed layers as a function of the Al or rather Ga concentrations

The surface morphology and the crystalline quality of the seed layers were investigated by atomic force microscopy (AFM) and X-ray diffraction (XRD) measurements, respectively, since these properties can influence the NW’s growth [20], as will be discussed later. For both types of seed layers (Al and Ga doped), we obtained a quite smooth surface. For the Al-doped layers, we observe a decrease of the average surface roughness from 2.4 to 1.3 nm with increasing Al concentration whereas for the Ga-doped layers, the roughness is typically between 2.0 and 2.6 nm (Fig. 1 a). Only for the layer with a concentration of *x*=3.5 at.%, we observed a reduction of the surface roughness down to 1.3 nm. The determined full width half maximum (FWHM) of the rocking curves as a function of the Al and Ga concentrations is shown in Fig. 1 b. For the undoped ZnO seed layer, we obtained a FWHM of about 0.8°. By doping the layer with Al, we observe an increase of the FWHM up to 2.4° for *x*=7 at.% whereas for the Ga doping, we do not observe a strong dependence on the Ga concentration. In this case, the FWHM was determined to be in the range of 0.8– 1.0°. Since the FWHM depends on the grain size [21, 22] and their tilting, we can estimate that the sizes and tilt of the grains for the Ga-doped seed layers are similar whereas for the Al-doped layers they increase with increasing of dopant concentration.

For the fabrication of the NWs, we use a high-pressure PLD process (HP-PLD). A detailed description of this technique is given in ref. [16]. In contrast to the conventional low-pressure PLD process, used for the fabrication of the seed layers, in the HP-PLD, an undoped ZnO target is used and the presence of a background gas flow is required in order to ensure a transport of the ablated particles from the target toward the substrate. Here, we used Ar as background gas (50 sccm) with a partial pressure of 150 mbar and varied the growth temperature in the range of *T*=400– 950^∘^C. As we will show later, the growth temperature has a strong impact on the growth of the NWs.

## Results and discussion

**Fig. 2:**
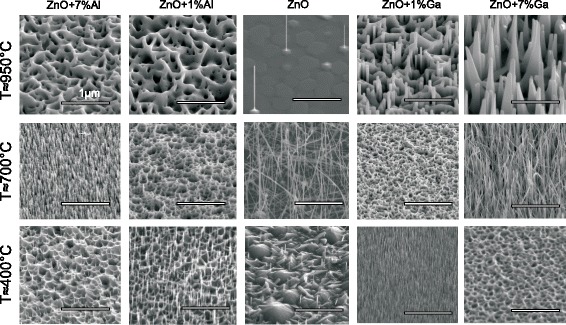
Scanning electron microscopy images of the nanostructures for Al- and Ga-doped ZnO seed layers for selected temperatures and concentrations

Scanning electron microscopy (SEM) images of the deposited nanostructures for selected temperatures and dopant concentrations of the seed layers are shown in Fig. 2. The SEM images for all doping concentrations and temperatures are shown in the Additional file 1: Figure S1 and Additional file 2: Figure S2. For the growth on the pure ZnO seed layer, a low density of vertically oriented NWs is obtained at *T*=950^∘^C as expected from previous results [16, 23]. For the seed layers used here, we obtained NWs with a diameter of about 70 nm and an aspect ratio of about 25. Note, it is also possible to fabricate NWs with larger diameters up to 600 nm [23–25]. By reducing the growth temperature down to *T*≈700^∘^C, the diameter of the NWs decreases to 16 nm and the aspect ratio increases up to 300. However, in contrast to the results obtained at *T*≈950^∘^C, these NWs are randomly oriented. A further reduction down to *T*≈400^∘^C leads to a suppression of the NW growth, and only very thin and short NWs with a diameter of 24 nm and aspect ratio of 6 were obtained.

By using Al- and Ga-doped ZnO seed layers, the NW growth with respect to the deposition temperature is different compared to that one observed for the undoped films. In the case of Al-doped layers, for the highest growth temperature of *T*≈950^∘^C, we observed a well-oriented growth of vertically aligned NWs only for Al concentration of *x*=3.5 at.% with a diameter of about 80 nm and an aspect ratio of 30 (see Additional file 1: Figure S1 and Additional file 2: Figure S2). For the other concentrations, the NW growth is suppressed and only the growth of a honeycomb-like structure is obtained. These honeycomb-like structures were not observed on the undoped seed layers. By reducing the temperature to *T*≈700^∘^C, NWs were obtained on the seed layer with a concentration of *x*=7 at.% only and the growth on the seed layers with *x*≤3.5 at.% is suppressed. Surprisingly, a further reduction of the growth temperature leads to a decrease of the Al concentration which supports the growth of NWs, i.e. at *T*≈600^∘^C, NWs are obtained for *x*=3.5 and *x*=2 at.% whereas for *T*≈400^∘^C, the growth is observed on the seed layers with *x*=1 and 2 at.%. Note, all NWs are well-oriented vertically, and for *T*≈400^∘^C the NWs have typically a diameter of *d*≤7 nm and an aspect ratio of 45. For the concentrations which does not support the growth of NWs at a given temperature, we observe the growth of a honeycomb-like structure.

The growth on the Ga-doped seed layers is slightly different at *T*≈950^∘^C compared to that one observed on the Zn _**x**_Al_**1−x**_O layers. At this growth temperature, the NW growth is suppressed for the seed layer with a doping concentration of *x*=3.5 at.%. However, for *x*=7 at.% an elongated pyramidal structure was obtained. For the other concentrations and growth temperatures, the results are similar to that one obtained on the Al-doped seed layers. Remarkably, on the Ga-doped seed layer with *x*=1 at.% at *T*≈400^∘^C a high density of ultrathin NWs with a diameter of *x*≤7 nm and an aspect ratio of about 100 were obtained.

An impact of the Al concentration on the NW growth was also observed by Käbisch et al. [26]. They attributed this behavior to a change of the surface polarity from an O-terminated surface of the undoped ZnO layer to a Zn-terminated one of the doped layers. In the last case, honeycomb-like structures were obtained instead of NWs. The observed growth of the honeycomb structure for the Al- and Ga-doped layers in our experiments would also indicate such a change of the surface polarity. In order to verify this change, we determined the polarity of the seed layers by using an etching method described by Mariano and Hanneman [27]. Accordingly, we etched the seed layers in a diluted HCl acid with a concentration of 1:100 for 30 s. The etching rate was found to be about 1 nm per second by using spectroscopic ellipsometry. Only for the undoped seed layer as well as for Al-doped layers with *x*=1 at.%, we found an O-terminated surface which manifested itself by a pyramidal structure after the etching whereas for the other seed layers we found a crater-like structure which indicates a Zn-termination. However, in contrast to the results obtained by Käbisch et al., we were able to grow NWs on the Zn-terminated seed layers even at high temperature (Fig. 2). Thus, the change of the polarity of the surface seems to be unlikely for the difference of the observed growth behavior.

The surface roughness and the crystal quality seem to be also unlikely for the observed behavior. On the one hand, from XRD measurements, we can conlude that the grain size is similar for all Ga-doped ZnO films whereas for the Al-doped ZnO films, the grain size increases with increasing Al concentration. However, the observed growth is similar for both kinds of seed layers and approximately the same NW diameter can be achieved on these seed layers which is in contrast to the results obtained by Ting et al. [28]. They observed that the diameter of the NWs increases with the grain size of the seed layer. On the other hand, neither an increase of the diameter of the NWs with decreasing surface roughness as reported by Ghayour et al. [20] nor another correlations were found. Note that a change of the surface roughness and of the grain size caused by the elevated temperature during the growth process can be excluded since XRD and AFM measurements performed on reference samples and annealed under deposition conditions do not show a significant change of the surface roughness and grain size.

A change of the observed morphology can be caused by a variation of the growth mechanism [18, 29]. A possible reason of such variation might be a change of the free energy given by *Δ* *F*=*F*_*m*_+*F*_*i*_−*F*_*s*_ [29, 30]. Here *F*_*m*_,*F*_*i*_, and *F*_*s*_ represent the free energy of the incoming material, the interface, and the surface, respectively. However, the experimental determination of the free energy components of the HP-PLD process is challenging.

**Fig. 3:**
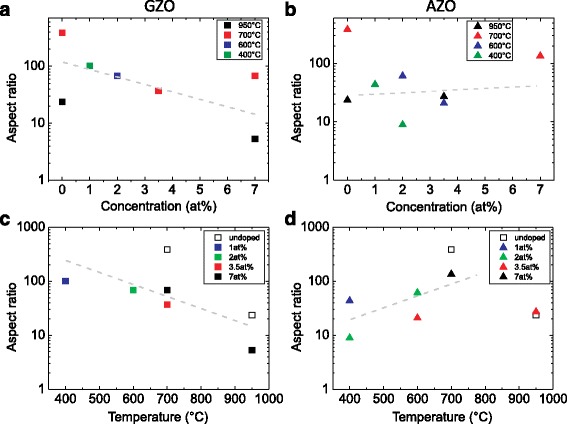
The aspect ratio of the NWs as a function of the doping concentration (**a**, **b**) and growth temperature (**c**, **d**) for the Ga-doped (**a**, **c**) and Al-doped seed layers (**b**, **d**). The *gray dashed lines* are guides for the eyes

**Fig. 4:**
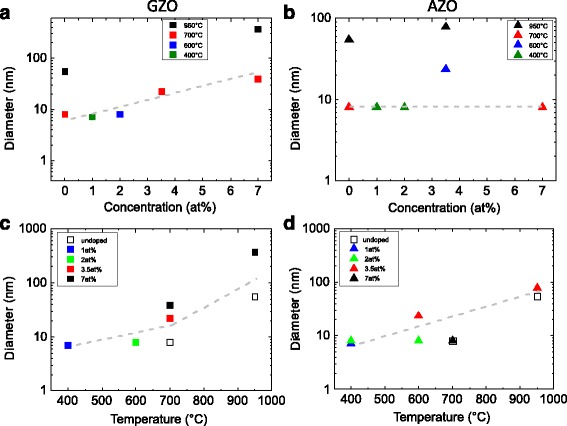
The diameter of the NWs as a function of the doping concentration (**a**, **b**) and growth temperature (**c**, **d**) for the Ga-doped (**a**, **c**) and Al-doped seed layers (**b**, **d**). The *gray dashed lines* are guides for the eyes

The aspect ratio and the diameter of the obtained NWs as a function of the doping concentration of the seed layers and the growth temperature are shown in the Figs. 3 and 4, respectively. The aspect ratio of the NWs grown on Ga-doped ZnO layers decreases with increasing doping concentration, whereas it decreases with increasing temperature. At the same time, an increase of the NW diameter with increasing Ga concentration and growth temperature is observed. For the growth on the Zn_**x**_Al_**1−x**_O seed layers, the situation is slightly different. The aspect ratio of the NWs is almost independent on the Al concentration and increases with the growth temperature. A comparison of the obtained aspect ratio and diameter for the Al- and Ga-doped layers exhibit that the Ga doping of the ZnO seed layer has a stronger impact on the NW growth than the doping with Al. This effect might be explained by the larger ionic size of Ga dopants which are probably more attractive for the deposited ZnO particles, i.e. the particles remain close to the surface of the seed layer and their motion toward the NW axis is reduced. Thus, the lateral growth rate is enchanced, i.e. the NW diameter increases, whereas the vertical growth is reduced, i.e. the NW length decreases. At the same time, the kinetic energy of the moving ZnO particles on the surface of the substrate strongly depends on the deposition temperature, which increases with increasing temperature. Moreover, such kinetic energy might be responsible for the lateral mobility and the lateral growth rate for both Al and Ga doping.

**Fig. 5:**
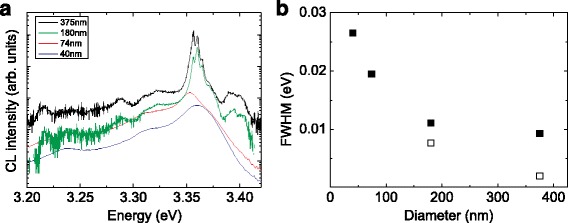
**a** CL spectra of ZnO NWs for different diameters. For a better clarity, the spectra were shifted vertically. **b** *Solid points*—the FWHM from entire emission as a function of diameter of the NWs, *empty points*—the FWHM from the single peaks for thick NWs

The optical properties of the NWs were investigated by cathodoluminescence (CL) at low temperature (*T*≈10 *K*). In order to avoid a signal from the seed layer, each sample was cut and the CL was done from the side of the sample. The NWs were excited by using an electron current of about 200 pA for the thick NWs (*d*≥180 nm) and 400 pA for the thin NWs. The enhancement of the current for the thin NWs was necessary due to their low photon emission. The emission of the NWs with *d*≤7 nm is not shown here since in this case, the observed luminescence cannot be distinguished from those originated from the seed layer. The emitted light was spectrally analyzed by a monochromator with a focal length of 320 mm and a grating of 600 lines/mm. The obtained CL spectra are shown in Fig. 5 a. For the thick NWs, we observed strong emission peaks around 3.36 eV as reported in literature which mainly originates from the recombination of donor-bound excitons [31]. For the most prominent one at *E*≈3.361 eV, we obtained a broadening of about 2 meV which is close to the value determined on bulk single crystals [32] and reflects the high crystalline quality of the NWs. With decreasing diameter, a strong increase of the broadening is observed from 2 meV for a diameter of 375 nm to 7.7 meV for a diameter of 40 nm. By reducing the diameter further, the observed peaks strongly overlap with each other and cannot be distinguished anymore. In this case, the increase of the broadening is also reflected by an increase of the broadening of the entire emission peak (Fig. 5 b). Responsible for the observed increase of the broadening might be the bending of the energy bands at the surface near region [33, 34] and thus the change of the electronic structure and emission characteristic. By reducing the diameter of the NWs, the surface-to-volume ratio strongly increases and thus the emission characteristic of the surface near region is dominant.

## Conclusions

To summarize, we have shown that the doping concentration of the Zn _**x**_Al_1−**x**_O and Zn _**x**_Ga_1−**x**_O has a strong impact on the NW growth. In doing so, we were able to tune the diameter by two orders and are able to grow vertically well-oriented ultrathin NWs. These ultrathin NWs are promising for the investigation of quantum effects of the electronic structure. Furthermore, these NWs can be achieved at temperatures of *T*≈400^∘^C which is suitable for the preservation of CMOS structures. The choice of the doping material of the seed layer influences also the morphology of the NWs. For the Zn _**x**_Ga_**1−x**_O, we observe an increase of the NW diameter and a decrease of the aspect ratio, whereas for the Zn _**x**_Al_**1−x**_O seed layers, these properties are almost constant. The optical properties of the NWs were investigated by CL experiments. For the thick NWs, we observe a broadening of the donor bound exciton of about 2 meV which reflects the high crystalline quality of the NWs. With decreasing diameter, the broadening of the emission peaks increases which we attribute to the strong increase of the surface-to-volume ratio for the thin NWs.

## Additional files

Additional file 1
**Figure S1.** Scanning electron microscope images of the nanostructures for Al-doped ZnO seed layers for all used temperatures and concentrations. (PDF 1249 kb)

Additional file 2
**Figure S2.** Scanning electron microscope images of the nanostructures for Ga-doped ZnO seed layers for all used temperatures and concentrations. (PDF 1105 kb)
