# Supplementary figures and images for: Low-Temperature PLD-Growth of Ultrathin ZnO Nanowires by Using ZnxAl1−xO and ZnxGa1−xO Seed Layers
Source: Nanoscale Res Lett. 2017 Feb 20;12:134. doi: 10.1186/s11671-017-1906-2 (PMC5318314; doi:10.1186/s11671-017-1906-2)

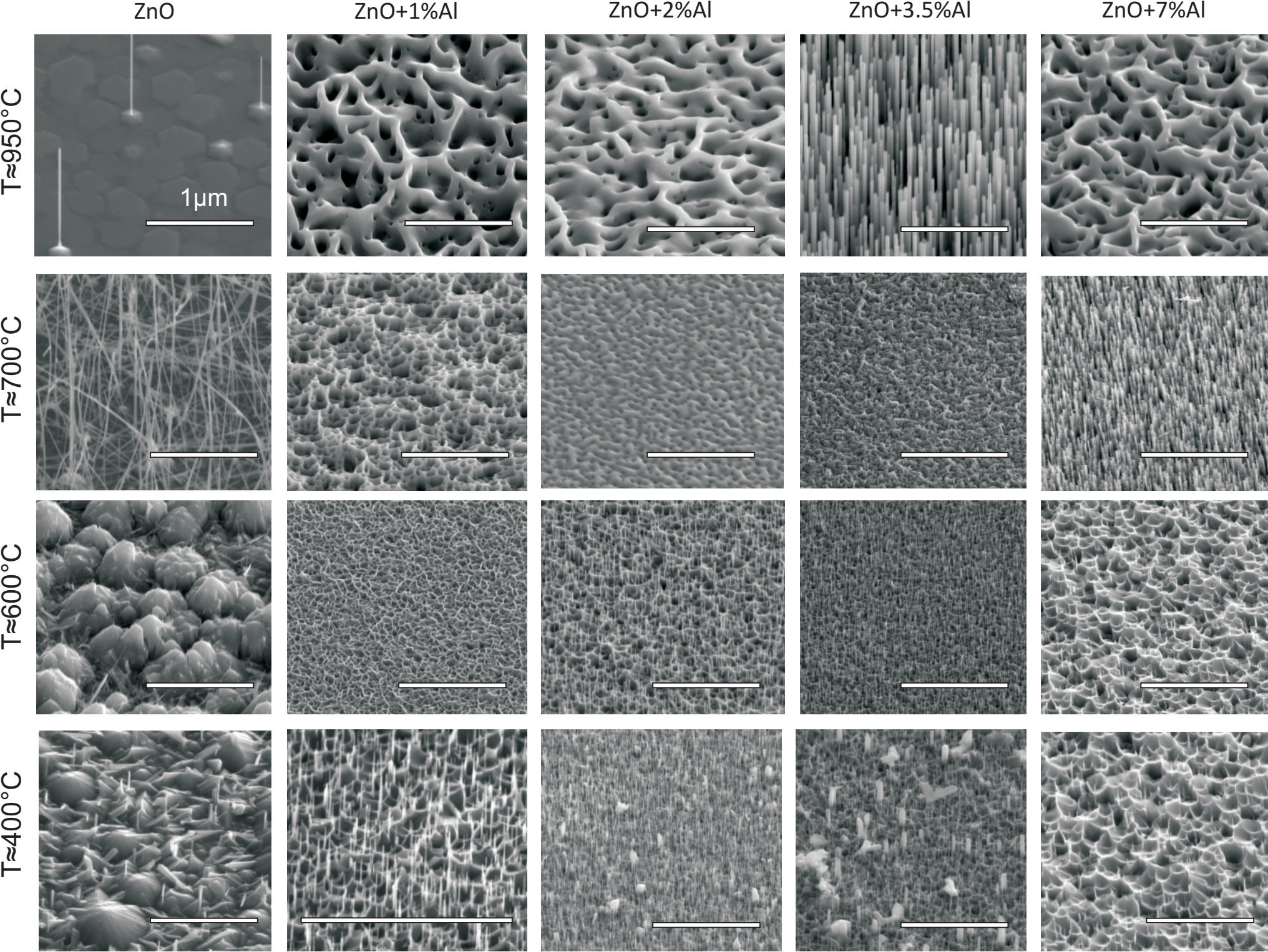

Supplement: Additional file 1 — Figure S1. Scanning electron microscope images of the nanostructures for Al-doped ZnO seed layers for all used temperatures and concentrations. (PDF 1249 kb) [file 11671_2017_1906_MOESM1_ESM.pdf]

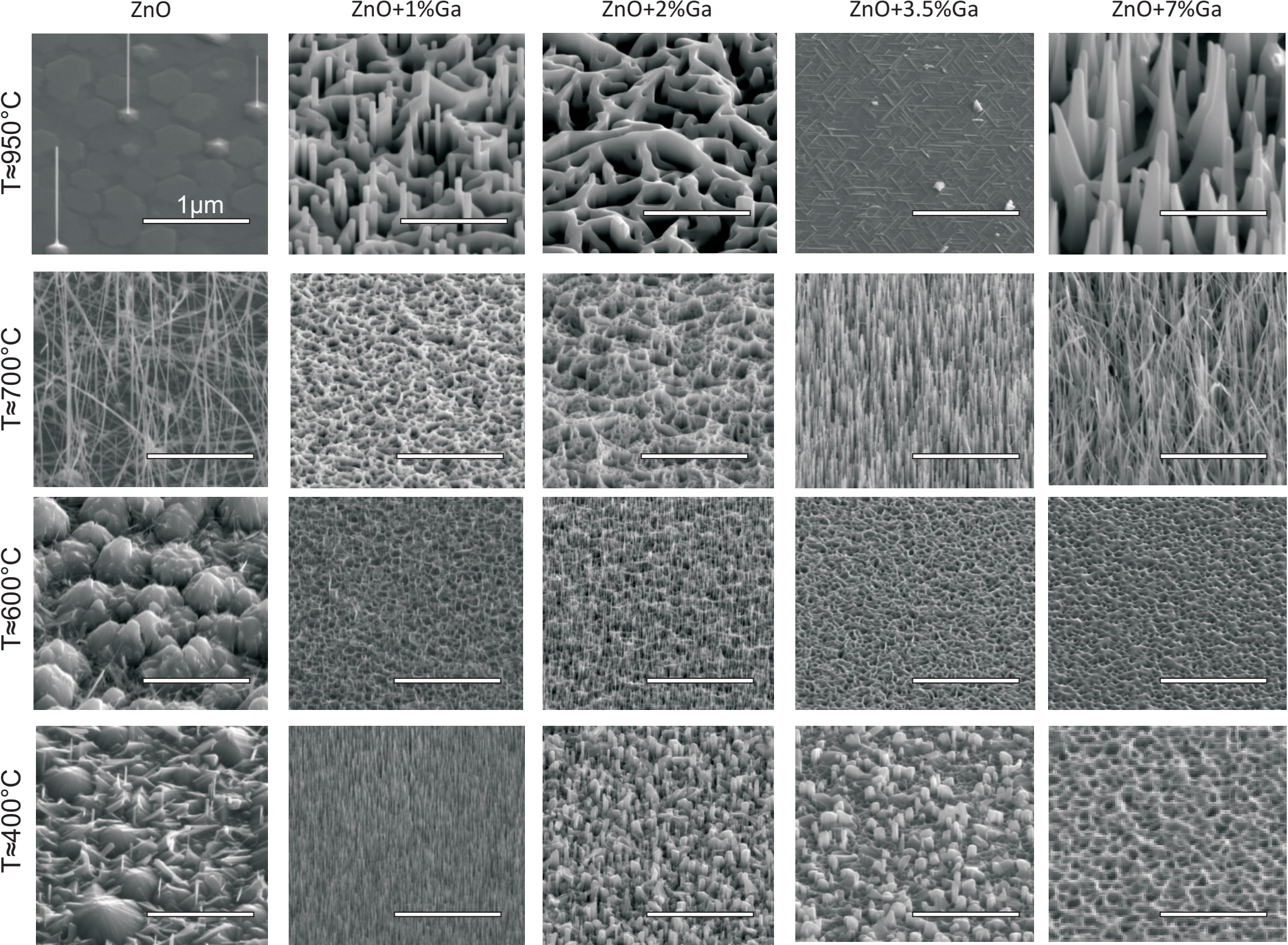

Supplement: Additional file 2 — Figure S2. Scanning electron microscope images of the nanostructures for Ga-doped ZnO seed layers for all used temperatures and concentrations. (PDF 1105 kb) [file 11671_2017_1906_MOESM2_ESM.pdf]
